# Supplementary material for: Revealing the benefit of eye motion for acuity under emulated cone loss
Source: bioRxiv. 2026 Mar 23:2026.03.19.712913. Preprint. [Version 1] doi: 10.64898/2026.03.19.712913 (PMC13042072; doi:10.64898/2026.03.19.712913)
Supplement: Supplement 1 [file NIHPP2026.03.19.712913v1-supplement-1.pdf]

bioRxiv preprint doi: https://doi.org/10.64898/2026.03.19.712913; this version posted March 23, 2026. The copyright holder for this preprint (which was not certified by peer review) is the author/funder, who has granted bioRxiv a license to display the preprint in perpetuity. It is made available under aCC-BY 4.0 International license.

# Supplementary Material for: Revealing the benefit of eye motion for acuity under emulated cone loss

Hannah K. Doyle[1], James Fong[1], Ren Ng[1], Austin Roorda[2]
[1]Department of Electrical Engineering & Computer Sciences, University of California, Berkeley, Berkeley, CA
[2]Herbert Wertheim School of Optometry & Vision Science, University of California, Berkeley, Berkeley, CA

bioRxiv preprint doi: https://doi.org/10.64898/2026.03.19.712913; this version posted March 23, 2026. The copyright holder for this preprint (which was not certified by peer review) is the author/funder, who has granted bioRxiv a license to display the preprint in perpetuity. It is made available under aCC-BY 4.0 International license.

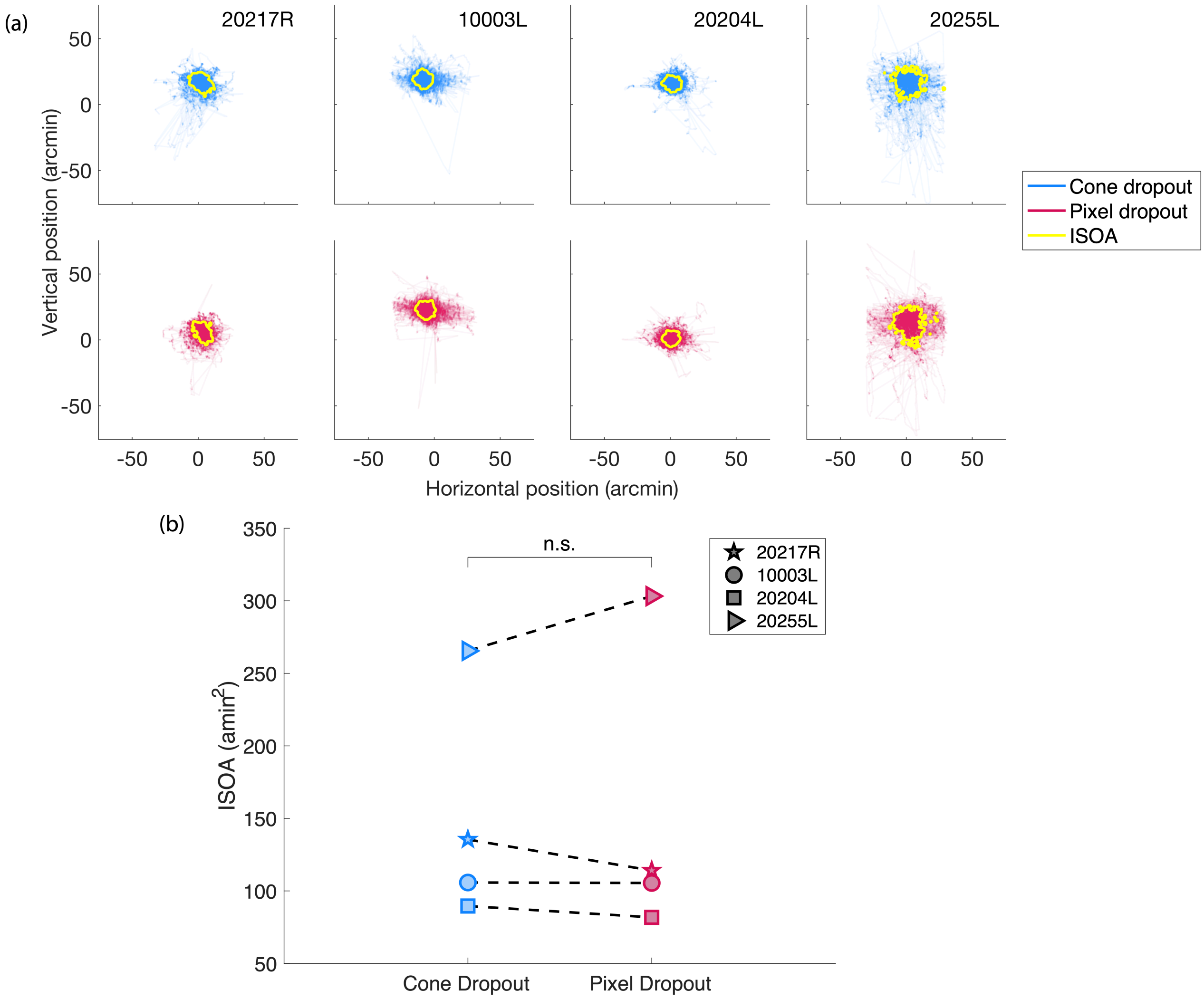


**Figure S1**. Iso-density contour area comparison between dropout conditions. (a) Eye motion traces are plotted for the cone dropout condition (blue) and pixel dropout condition (red) in the experiment varying stimulus duration for 4 subjects as indicated in the upper right corner of each column. The iso-density contour line containing 68% of the eye motion data is plotted on top of the eye motion traces. The apparent cutoff for some traces in the horizontal dimension arises when the subject's eye moves outside of the trackable region. (b) The iso-density contour area (ISOA) enclosed by the contours is plotted for the cone dropout (blue) and pixel dropout (red) conditions. Each symbol indicates a different subject. No significant difference was found between ISOA under the cone dropout and pixel dropout conditions.
